# Transcriptional correlates of the pathological phenotype in a Huntington’s disease mouse model

**DOI:** 10.1038/s41598-019-55177-9

**Published:** 2019-12-10

**Authors:** Andrea Gallardo-Orihuela, Irati Hervás-Corpión, Carmen Hierro-Bujalance, Daniel Sanchez-Sotano, Gema Jiménez-Gómez, Francisco Mora-López, Antonio Campos-Caro, Monica Garcia-Alloza, Luis M. Valor

**Affiliations:** 1 Instituto de Investigación e Innovación en Ciencias Biomédicas de la Provincia de Cádiz (INiBICA), Cádiz, Spain; 2 Unidad de Investigación, Hospital Universitario Puerta del Mar, Av. Ana de Viya 21, 11009 Cádiz, Spain; 3 Área de Fisiología, Facultad de Medicina, Universidad de Cádiz, Plaza Fragela, 11003 Cádiz, Spain; 4 Servicio de Inmunología, Hospital Universitario Puerta del Mar, Av. Ana de Viya 21, 11009 Cádiz, Spain

**Keywords:** Transcription, Huntington's disease

## Abstract

Huntington disease (HD) is a fatal neurodegenerative disorder without a cure that is caused by an aberrant expansion of CAG repeats in exon 1 of the huntingtin (*HTT*) gene. Although a negative correlation between the number of CAG repeats and the age of disease onset is established, additional factors may contribute to the high heterogeneity of the complex manifestation of symptoms among patients. This variability is also observed in mouse models, even under controlled genetic and environmental conditions. To better understand this phenomenon, we analysed the R6/1 strain in search of potential correlates between pathological motor/cognitive phenotypical traits and transcriptional alterations. HD-related genes (e.g., *Penk*, *Plk5*, *Itpka*), despite being downregulated across the examined brain areas (the prefrontal cortex, striatum, hippocampus and cerebellum), exhibited tissue-specific correlations with particular phenotypical traits that were attributable to the contribution of the brain region to that trait (e.g., striatum and rotarod performance, cerebellum and feet clasping). Focusing on the striatum, we determined that the transcriptional dysregulation associated with HD was partially exacerbated in mice that showed poor overall phenotypical scores, especially in genes with relevant roles in striatal functioning (e.g., *Pde10a*, *Drd1*, *Drd2*, *Ppp1r1b*). However, we also observed transcripts associated with relatively better outcomes, such as *Nfya* (CCAAT-binding transcription factor NF-Y subunit A) plus others related to neuronal development, apoptosis and differentiation. In this study, we demonstrated that altered brain transcription can be related to the manifestation of HD-like symptoms in mouse models and that this can be extrapolated to the highly heterogeneous population of HD patients.

## Introduction

Huntington’s disease (OMIM #143100) is the most prevalent autosomal dominant polyglutamine (polyQ) disorder (5–10 cases per 100,000 inhabitants worldwide), and it is caused by an aberrant expansion of polymorphic trinucleotide CAG repeats (>36) in exon 1 of the huntingtin (*HTT*) gene^1^. Despite intensive efforts to elucidate the etiopathological mechanisms triggered by both the expression of a misfolded mutant HTT fragment (mHtt) and the loss of a functional allele, no effective cure is available to patients, who suffer progressive impairments in mental and motor functions until death.

To improve and personalize clinical counseling, there is a need for valuable biomarkers to monitor the health status of individuals, assess the dosing, efficacy and safety of potential therapeutic approaches and minimize uncertainty in clinical decision-making. This is especially relevant in HD, as there is increasing evidence of a long-lasting presymptomatic stage with underlying pathological processes^2^; the number of CAG repeats, although inversely correlated with the age of disease onset^3,4^, is unable to explain the progression and manifestation of certain symptoms^5–7^. In the search for prognostic biomarkers, peripheral blood has been the preferred source^8–13^. Among the strategies for biomarker screening, the exploration of RNA molecules is advantageous because it offers a comprehensive and genome-wide repertoire of candidates (both coding and non-coding species) in a cost-effective manner. In addition, potential RNA-based markers may be directly linked to the initial stages of disease since transcriptional dysregulation is an early event that occurs prior to cell death, mHtt aggregation and mitochondrial dysfunction in cellular preparations and is clearly evident in animal models with minimal phenotypical alterations, occurring in both the brain and peripheral tissues^14,15^. However, the peripheral biomarkers that have been proposed thus far have been shown to be substantially inconsistent across the examined human cohorts due to a combination of factors^16^, including those related to the environmental and genetic variability of unsupervised human populations as important modulators of the complex HD symptomatology. Thus, intensive efforts have focused on identifying genetic modifiers of the age of onset and progression of motor impairment^17,18^.

To gain insights into the interindividual variations in the manifestation of HD symptoms, the analysis of mouse models is pertinent, as these variations can also be observed under controlled environmental conditions and on a controlled genetic background, although expectedly to a much lesser extent than in humans. Transcriptional dysregulation is relatively well preserved between mouse and human brains^19,20^; therefore, the resulting candidates may be extrapolated to patients. Previous reports have found correlations between gene expression and stages of the disease in both slow and rapidly progressive models of HD^21,22^, but to our knowledge, no study has established this type of correlation on an individual basis. Here, we describe that even slight transcriptional and phenotypical variations can show certain associations in a tissue-dependent manner in environmentally and genetically controlled R6/1 mice.

## Results

### Phenotypical and transcriptional characterization of the early symptomatic R6/1 strain

**Figure 1:**
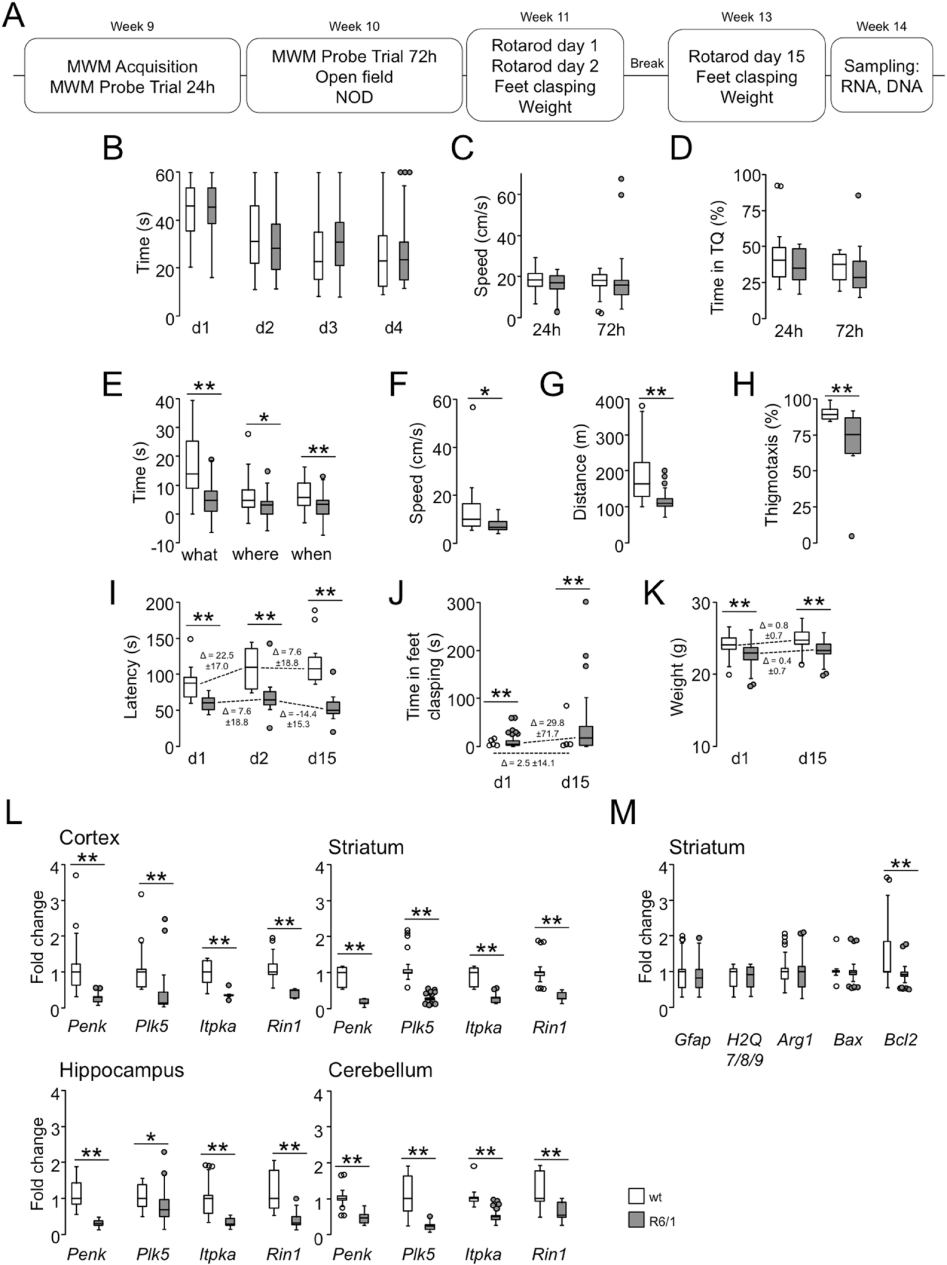
Behavioural and gene expression analysis of early symptomatic R6/1 mice. (**A**) Timeline of the behavioural test battery and the sampling of R6/1 mice and their wild-type littermates. MWM, Morris water maze; NOD, novel object discrimination. *B-D*, The Morris water maze task did not reveal differences between genotypes in performance in the acquisition phase (**B**) or in the swimming speed (**C**) or time spent in the target quadrant (TQ) in the probe trials (**D**). (**E**) R6/1 mice exhibited significant impairments in the three components (“what”, “where” and “when”) of the NOD task. (**F**–**H**) Spontaneous activity was measured in the open field test, and differences in the speed (**F**), distance travelled (**G**) and the time spent in the border area (*H*) were identified. (**I**) R6/1 animals exhibited significant impairments in the accelerating rotarod task. Motor learning (Δ = day 2 − day 1) was also affected. (**J**) R6/1 mice exhibited obvious feet clasping. (**K**) Mutant mice were unable to gain weight, unlike their wild-type littermates. *I-K*, Progression rates (Δ = week 13 − week 11) for accelerating rotarod performance (**I**), feet clasping (**J**) and weight gain (**K**). (**L**) Genes of a consistent HD transcriptional signature (*Penk* (proenkephalin), *Plk5* (polo-like kinase 5), *Itpka* (inositol trisphosphate 3-kinase A), and *Rin1* (Ras and Rab interactor 1)) were downregulated in the prefrontal cortex, striatum, hippocampus and cerebellum of R6/1 mice, as determined by RT-qPCR assays. (**M**) Except for *Bcl2* (B-cell lymphoma 2), the degeneration-related genes *Gfap* (glial fibrillary acidic protein), *H2Q7/8/9* (histocompatibility 2, Q region locus 7/8/9), *Arg1* (arginase 1), and *Bax* (Bcl-2-associated X) were not affected. In all the panels: n wt = 24, n R6/1 = 29; *P < 0.05; **P < 0.005 between genotypes; Mann Whitney U-test. The data are expressed as whisker-and-box plots.

To expand on the phenotypical characterization of the R6/1 strain on a pure C57/BL6J background, which was initiated in a previous study^23^, a battery of behavioural tests was conducted at early symptomatic stages (from 9 to 13 weeks) to assess the cognitive and motor performances of 24 wild-type and 29 mutant males (see scheme in Fig. 1A and Materials and Methods for a detailed description of the animals). Based on previous experiences^24^, we started with cognitive assessments to minimize the impact of the motor component of the pathology on the data. In the Morris water maze task (week 9), mutant mice did not show differences compared to their wild-type littermates (Fig. 1B–D), but cognitive impairment was more evident in the novel object discrimination (NOD) task, in which mutant mice were highly inefficient at remembering familiar objects and both spatial and temporal changes (Fig. 1E). Although the spontaneous activity of these animals was lower than that of their wild-type littermates at week 10 (Fig. 1F,G), reduced thigmotaxis (Fig. 1H) indicated alterations in motivation in addition to motor deficits. Motor impairment was clearly evident in the accelerating rotarod task, as mutant mice showed shorter latencies and diminished learning capabilities (Fig. 1I). Besides, mutant mice exhibited feet clasping (Fig. 1J) and weight loss (Fig. 1K), which both are characteristic of HD mice. These phenotypical traits were measured in 11- and 13-week-old animals, showing a trend toward the exacerbation of the differences between the genotypes in only two weeks (Fig. 1I–K, compare the Δ values of R6/1 and wild-type mice).

During these experiments, we observed a certain degree of variability among individuals, with a median coefficient of variation (CV) of 45% (interquartile range = 28–59%) and 44% (interquartile range = 32–106%) across the tasks for wild-type and mutant mice, respectively. To explore the possible causes of such variability in these animals, we obtained samples of the prefrontal cortex, the striatum, the hippocampus and the cerebellum from the mice tested on the behavioural battery five days after the last test (week 14) for the sequential isolation of total RNA and gDNA. Then, we evaluated the degree of transcriptional dysregulation in the samples by examining transcripts consistently affected in a variety of brain areas in mouse models of HD: *Penk*, *Plk5*, *Iptka* and *Rin1*^20,23,24^. We confirmed the prominent downregulation of these genes in the four brain areas collected from the R6/1 mice compared to their wild-type littermates with RT-qPCR (Fig. 1L), as reported in our previous work. In contrast, the expression of a second set of genes related to inflammatory processes and degeneration (*Gfap* for gliosis, *H2Q7/8/9* for major histocompatibility complex type Ib, *Arg1* for M1 microglial activation and *Bax* for the promotion of apoptosis) was not different between the genotypes in the striatum, the area that is the most sensitive to mHtt expression (Fig. 1M); the exception was *Bcl2*, which can antagonize the function of *Bax*^25^ but which role in HD is still under debate^26^. In conclusion, the R6/1 mice used in this study were significantly impaired at the behavioural and transcriptional levels in early stages of the disease without a prominent influence of cell death processes.

### Correlative patterns between phenotype and gene expression

**Figure 2:**
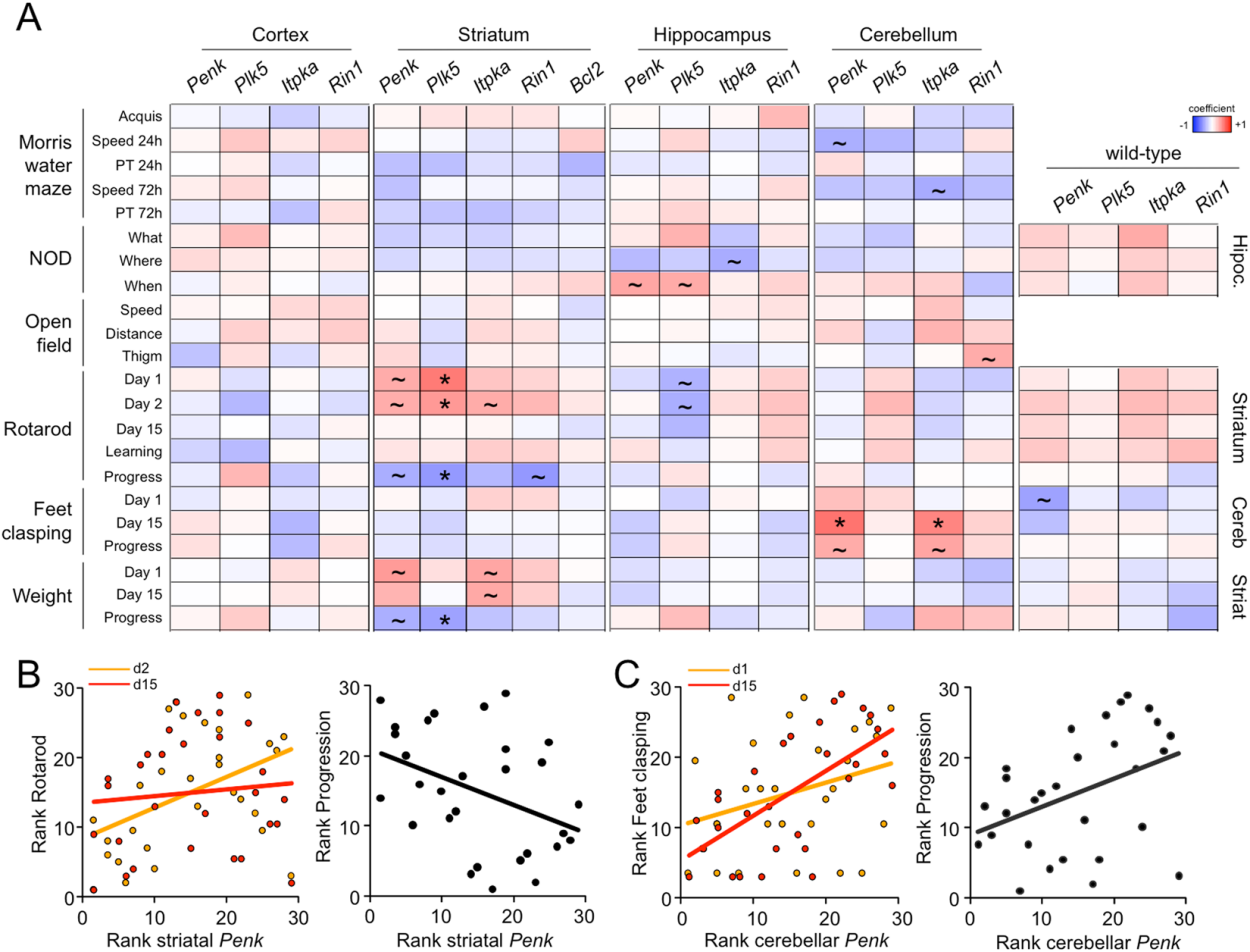
Transcriptional and behavioural variabilities in R6/1 mice are mildly correlated in a tissue-dependent manner. (**A**) Summary of the Spearman coefficient values between the analysed phenotypical traits and gene expression levels. Significant correlations (~unadjusted *P* < 0.05; *adjusted *P* < 0.1, linear regression t-test) were observed between striatal gene expression and rotarod and weight, between hippocampal gene expression and performance on the NOD task and between cerebellar gene expression and feet clasping in R6/1 mice (n = 29). These correlations were not observed in the wild-type animals (n = 24). PT, probe trial; Thigm, thigmotaxis. (**B**) The correlation between striatal *Penk* transcript variations and performance on the accelerating rotarod was reduced at the age of 13 weeks. Left panel, the latency to fall off the rod and *Penk* expression in mutant mice ranked from the highest to the lowest values for each time point; week 11 (d2) and week 13 (d15). Right panel, the association between progression rate (week 13 − week 11) and the striatal *Penk* expression level. (**C**) The correlation between cerebellar *Penk* transcript variations and feet clasping was increased at the age of 13 weeks. The panels are displayed as in *B*.

To investigate whether the transcriptional dysregulation of the HD signature was an indicator of the degree of behavioural dysfunction, we ranked the animals according to their values obtained for each parameter from less to more affected to establish potential correlations between phenotypical and transcriptional outcomes using the Spearman’s rank-order coefficient (see Materials and Methods for further details). Although we analysed the same deregulated genes across the four brain areas, distinctive tissue-specific patterns were observed: meanwhile there was no evident correlation between behaviour and gene expression changes in the prefrontal cortex, the changes of *Penk*, *Plk5* and/or *Itpka* in the other areas was positively correlated with performance in various tasks: downregulation in the striatum was correlated with poor performance in the accelerating rotarod and/or weight loss, downregulation in the hippocampus with poor performance in the NOD task, and downregulation in the cerebellum with the severity of feet clasping (Fig. 2A). Certain individuals belonging to the same litters tended to rank similarly, suggesting subtle inherited/environmental differences between litters/cohorts that were not entirely controlled in our experimental design (Supplementary Fig. S1). In any case, these correlations were not observed in wild-type mice. Of note, positive correlations with striatal gene alterations at 11 weeks of age were not evident at 13 weeks, closer to the time of sampling; as a result, the progression of motor discoordination and weight loss (calculated as the Δ between week 13 and week 11) were negatively correlated with transcriptional dysregulation (“Progress” in Fig. 2B). This phenomenon can be tentatively explained by the faster worsening of HD-like phenotypes in animals that were initially less affected by mHtt expression at 11 weeks of age, which reduced (but did not abrogate) the behavioural variability between individuals (Fig. 2B). In contrast, the correlations with cerebellar changes were more pronounced at the later time point (Fig. 2C), indicating that HD symptoms can develop differently in these mutant mice.

**Figure 3:**
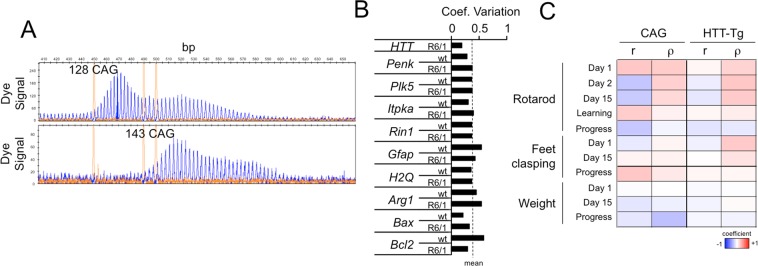
Behavioural variability in R6/1 mice is not attributable to the number of CAG repeats or transgene expression in the striatum. (**A**) Representative electrophoregrams of DNA fragment analysis of a mutant mouse from the first cohort (upper panel) and last cohort (lower panel) showing an increase in the number of CAG repeats. The sharp yellow peaks are the molecular weight standards. (**B**) Compared to other genes, the expression of the R6/1 transgene in the striatum was relatively stable across the mutant mice. Coeff.Variation = coefficient of variation (s.d./mean). (**C**) Summary of the Pearson (r) and Spearman (ρ) coefficient values indicating no correlation between phenotypical traits and the number of CAG repeats/transgene expression (n R6/1 = 29).

These results suggested that alterations in gene expression and phenotype are linked. However, other phenomena, such as the number of CAG repeats and mHtt transgene expression, might be more relevant to behaviour and hence more strongly correlated. We focused on the striatum, the main area affected in HD that also shows the highest genetic instability of CAG repeats^27,28^, for our subsequent experiments. After we determined the number of repeats in the isolated gDNA of each individual, we observed a slight but significant increase in the number of CAG repeats (~10) over the span of the behavioural assays (Fig. 3A). However, this increase was not sufficient to have a profound impact on phenotype, as revealed by the absence of a meaningful correlation between the number of repeats and impairments in striatal-dependent phenotypes (Fig. 3C). Expression of the transgene was the most stable among the genes tested in our study (Fig. 3B), and no correlation with phenotype was observed (Fig. 3C). Based on these observations, we found that the level of transcriptional dysregulation of certain genes can be associated to the degree of impairment for specific phenotypical traits in the R6/1 strain, without a profound influence of the number of CAG repeats and mHtt transgene expression in defining the interindividual variability in the mice of our study.

### Transcriptional dysregulation is exacerbated in R6/1 mice with a worse phenotype

**Figure 4:**
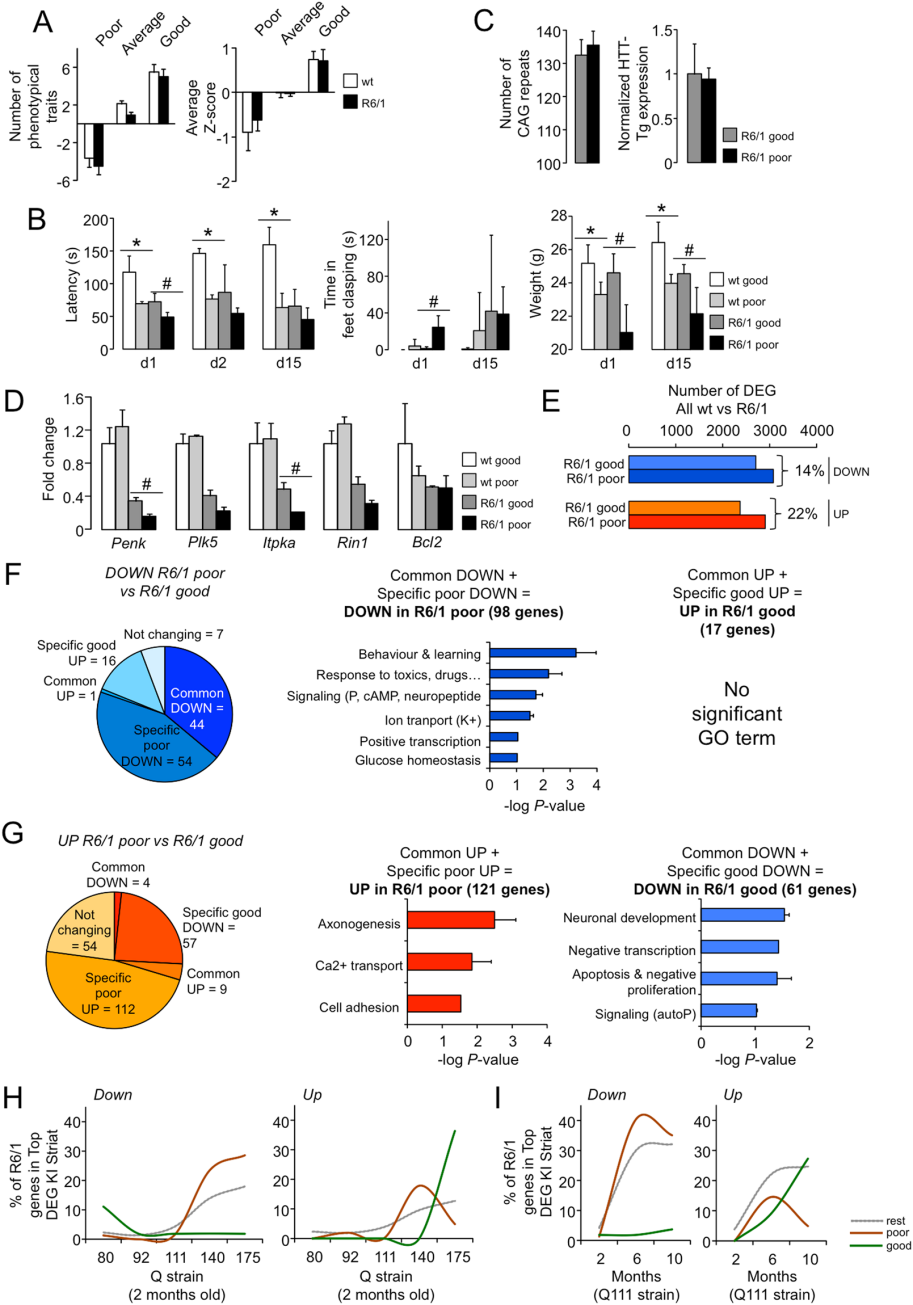
HD transcriptional dysregulation is exacerbated in a worse pathological phenotype. (**A**) Summary of Z-score calculations for classifying the mice according to their phenotype during weeks 11 and 13: “poor”, “good” and “average”. Left panel, the number of phenotypical traits with positive or negative Z-scores for the three categories of mice. Right panel, Z-score values represented as the mean ± s.e.m. (**B**) Accelerating rotarod performance (left), feet clasping (middle) and weights (right) across the four groups of animals according to genotype (wt, R6/1) and phenotype (good, poor); n = 4 in each group. (**C**) No difference in the number of striatal CAG repeats or mHtt transgene expression between R6/1 “good” and “poor” animals was observed. (**D**) The expression of genes belonging to the HD signature across the four groups of animals. In contrast to phenotype, differences were only observed in mutant mice by RT-qPCR. *P < 0.05; **P < 0.005 between “good” and “poor” wild-type mice; #*P* < 0.05; ##<0.005 between “good” and “poor” R6/1 mice; Mann Whitney U-test. The data are expressed as the mean ± s.d. *E*, The number of differentially expressed genes (DEGs) related to the wild-type striatum (both downregulated and upregulated) was higher for the R6/1 “poor” striatum compared to the R6/1 “good” striatum. (**F**,**G**) Pair-wise comparisons between R6/1 “poor” and “good” striatal transcriptomes identified four types of genes based on the direction of change (downregulated or upregulated) and the group of mutants in which they were more altered (“good” or “poor”). Each set of genes is represented by the results of GO enrichment analysis (*P* < 0.1, DAVID), except for the upregulated genes in the R6/1 “good” striatum for which no significance was retrieved. The data are expressed as the mean ± s.e.m of log *P* values across the terms in each category. Common, common DEGs to pair-wise comparisons between wild-type and R6/1 “poor” and between wild-type and R6/1 “good”; Specific, DEGs only appearing in one comparison; Not changing, DEGs between R6/1 “poor” and “good” but not altered between wild-type and R6/1. (**H**,**I**) The presence (expressed as %) of the four sets of genes identified in (**F**,**G**) among the top DEGs between KI HD mice with expanded CAG repeats and with non-pathological range number of repeats (Q20) at the indicated ages. Two additional sets of genes (genes that are dysregulated in HD but are not different between the R6/1 “poor” striatum and the R6/1 “good” striatum) were also included (“rest”). The downregulated “poor” genes and upregulated “good” genes exhibited the most progressive behaviour compared to that of the other sets, both through analysing the effect of the CAG length (**H**) and age (**I**) on phenotype worsening.

To better understand the relationship between phenotype and transcription in R6/1 mice, we selected those animals of the same age (14 weeks) that were differentially affected by mHtt expression at the behavioural level using an independent approach to the correlation analysis. To simplify this approach, we focused on phenotypical traits measured three weeks before sample collection to identify the wild-type and mutant animals that deviated the most from the average in terms of rotarod performance, duration of feet clasping and weight using Z-scores (see Materials and Methods for further details). The four mice of each genotype with the highest and lowest Z-scores were classified as “good” and “poor” performers, respectively (Fig. 4A,B). Mutant mice in these two categories did not show differences in the number of CAG repeats or mHtt transgene expression (Fig. 4C) but were prone to exacerbate the downregulation of the consistent HD signature analysed in the qRT-PCR assays; this trend was not observed in wild-type animals (Fig. 4D).

To obtain a complete picture of the gene expression changes associated with worse phenotype/performance, striatal RNA was processed for RNA-seq assays to determine the differential gene expression between “good” and “poor” mice of each genotype. Whereas no single differentially expressed gene (DEG) between both types of wild-type animals was detected, 233 were upregulated and 121 genes downregulated in the striatum of “poor” mutant mice compared to “good” mutant mice (adjusted *P*-value <0.05). Based on the apparent lack of relevant differences among wild-type mice, we pooled the wild-type datasets to increase the statistical power of the pair-wise comparisons for each R6/1 group. Then, we investigated the association between the severity of the phenotype and HD transcriptional dysregulation. Compared to mutant mice that exhibited less phenotypical impairment, mutant mice that showed a worse phenotype were more affected at the level of striatal gene expression (Fig. 4E). Compilation of these results defined four sets of striatal genes that were significantly altered between R6/1 “poor” and R6/1 “good” mice but also between wild-type animals to ensure that we studied changes associated with the HD transcriptional dysregulation (Fig. 4F,G). These sets of genes were: (i) those that were more significantly downregulated in R6/1 “poor” animals, which included *bona fide* HD-related genes, such as the proenkephalin *Penk*, the phosphodiesterase *Pde10a*, the dopamine receptors *Drd1* and *Drd2*, and the phosphorylation regulator DARPP-32 (*Ppp1r1b*) among other neuronal genes, as evidenced by the Gene Ontology (GO) enrichment analysis with the terms “Behaviour & learning”, “Neuropeptide”, and “Potassium transport”; (ii) those that were more significantly downregulated in R6/1 “good” animals, which included genes involved in neuronal development and apoptosis, such as the bilateral polymicrogyria-associated *Adgrg1* (aka *Gpr56*), the inhibitor of cyclin-dependent kinases *Cdkn1a* (aka p21), the inhibitor of the mTOR signalling pathway *Ddit4*, the fibroblast growth factor receptor *Fgfr3*, and the schizophrenia and bipolar disorder-associated gene *Mdga*; (iii) those that were more significantly upregulated in R6/1 “poor” animals, which were mainly related to axonogenesis, Ca^2+^ regulation and cell adhesion and included the voltage-calcium sensitive channel *Cagna1g*, the cadherin *Cdh2*, the Down syndrome adhesion molecule *Dscam* and the axonal midline guidance gene *Slit1*; and (iv) those that were significantly upregulated in R6/1 “good” mice, such as *Nfya*, a subunit of the transcription factor NF-Y. All DEGs are shown in Supplementary Table 1.

Taking profit of the existing longitudinal transcriptomics study conducted in HD knock-in (KI) mice expressing different lengths of polyQ^21^, we validated the association between the newly defined sets of genes with conditions linked to phenotypical worsening. To this end, we first retrieved the top DEGs in the pair-wise comparisons between CAG-expanded strains (Q80, Q92, Q111, Q140 or Q175) and control mice (Q20) at different ages (2, 6 and 10 months). Then, we calculated the percentage of genes more affected in R6/1 “good” or “poor” mice amongst the top DEGs in the same direction of change in the KI striatum. Other sets of genes associated with HD transcriptional dysregulation but not significantly different between R6/1 “good” and “poor” mice (thereafter referred to as the “rest”) were also included in the analysis for comparative purposes. Regarding downregulated genes, both the “rest” and R6/1 “poor” genes showed a progressive increase in the percentage of top DEG in strains with longer stretches of CAG repeats (Fig. 4H) and in aged animals (Fig. 4I). However, the relative presence of “poor” genes amongst the top DEG in KI mice was always superior, suggesting a stronger relationship with phenotype worsening. In contrast, the downregulated genes in R6/1 “good” mice did not exhibit a progressive pattern (Fig. 4H,I left panels). The opposite behaviour was observed for upregulated genes; whereas the overrepresentation of R6/1 “good” genes were mostly observed in comparisons with older mice or animals with the highest number of CAG repeats, the R6/1 “poor” genes showed a transient profile (Fig. 4H,I right panels). The interpretation of the latter result was not straightforward unless we assume that the upregulated R6/1 “good” genes play a protective role against mHtt expression and are intensively activated in severe stages of the disease. In this regard, *Nfya* mRNA has been reported to be upregulated as part of a homeostatic mechanism for restoring the decreased protein levels of NF-Y in HD models^29^, an observation that we replicated in our R6/1 colony (Supplementary Fig. S2). The potential relevance of this CCAAT-binding factor to neurodegeneration was determined by conditionally knocking out *Nfya* in forebrain postmitotic neurons^30^. However, the impact of this phenomenon was difficult to discern at the level of known NF-Y target genes such as *Hsp90b1* (*aka Gpr94*) which was regulated in the opposite direction in our gene expression analysis, and its promoter retained the capability to bind both NF-Y_A_ and NF-Y_B_ subunits in the R6/1 striatum (Supplementary Fig. S2).

Finally, the retrieval of DEGs in the RNA-seq analysis enabled us to widen the correlative analysis of Fig. 2 by including more genes related to worse HD phenotype. However, we only identified a single potential association between transcriptional dysregulation and particular symptoms in *Gabrd*, another gene belonging to the consistent HD signature (Supplementary Fig. S3). Overall, we used an alternative approach to demonstrate that general worsening of the HD phenotype partially exacerbated HD-linked transcriptional dysregulation in parallel to a putative activation of homeostatic responses. However, this gene expression reprogramming did not necessarily explain the outcome of specific phenotypical traits.

## Discussion

In this study, we provided initial evidence as a proof of concept regarding putative correlations between transcriptional dysregulation and phenotypical impairment in HD. Pathological phenotypes were not correlated with degenerative markers (which were not profoundly altered in R6/1 mice compared to wild-type mice, as revealed by RT-qPCR and RNA-seq) or the number of CAG repeats. The latter case was surprising, considering the significant expansion of repeats across the duration of this study; however, it can be argued that such expansion is not sufficient to deeply influence specific phenotypical traits in the context of an extremely long stretch of CAG repeats. Of note, the evaluation of genes that were altered across multiple brain areas (i.e., those that belong to a consistent HD signature) revealed the association of tissue-dependent gene expression variations with particular pathophenotypical traits. Performance on the accelerating rotarod is known to be linked to striatal function^31,32^, and accordingly, we found correlations between striatal gene expression patterns and latency to fall off the rod. We also observed an association between feet clasping and cerebellar gene expression, which is in agreement with the manifestation of this trait in several mouse mutants with neurological conditions in which the cerebellum is affected by genetic manipulation^33–35^. The association between hippocampal gene expression and scores on the NOD task can be explained by the fact that this paradigm involves spatial learning and recognition memory, which requires the short-term participation of hippocampal circuitry^36^. More intriguing is the association between the striatum and weight loss. The cause of weight loss in HD patients is unknown, but the most likely contributing factors are a combination of hyperactivity of the sympathetic nervous system, energy expenditure and hypermetabolism, and swallowing difficulties. Although extensive research has been conducted on the role of the hypothalamus^37^, recent work has identified novel actions of the striatum in the regulation of food intake, likely taking part in a motivational mechanism underlying rewarding behaviour and the anticipation of food^38^. Whether this behaviour is sufficiently impaired in HD to explain weight loss remains to be determined. Overall, these data indicate that altered gene expression patterns can be helpful in ascribing preferential brain regions to phenotypical alterations using advanced cellular fractionation procedures and analytical tools without the requirement of housekeeping gene normalization. With the resulting information, it would be feasible to design selective therapeutic approaches in mouse models by targeting neural populations that are implicated in particular symptoms such as chorea and depression-like behaviours, to cite some relevant examples of symptoms that are clinically managed in patients.

Such approach will be also useful to better dissect the temporal course of molecular and behavioural events, as exemplified by the dysregulation of neuropeptides in HD. Among the correlated genes, *Penk* is the most well known in HD as its downregulation is considered a marker of neuronal dysfunction in the striatum in early stages of HD^39,40^. This gene encodes a precursor of enkephalins, which are opioid neuropeptides with neuroprotection properties^41^ that are mainly present in D2R-expressing striatopallidal medium spiny neurons (MSNs) in the indirect pathway of movement. These MSNs are thought to degenerate earlier than those in the direct pathway and thus can better explain the onset of chorea due to the inadequate activation of cortical areas^42^. The expression of other neuropeptides, such as *Pdyn*, which produces dynorphin, β-endorphin and others, and *Tac1*, which produces substance P, neurokinin A and others, were found to be linked to a worse HD phenotype according to the RNA-seq analysis. These neuropeptides are more related to D1R-expressing nigrostriatal MSNs in the direct pathway, the impairment of which seems to be linked to late-onset motor abnormalities such as akinesia and dystonia^42^.

Nonetheless, the transcription-phenotype association cannot be explained using a simplistic model of exacerbation of HD transcriptional dysregulation. Although it is true that the RNA-seq analysis conducted on striatal RNA from “good” and “poor” performers showed more pronounced changes in both directions (upregulation and downregulation), most of these changes were not correlated with specific HD-like symptoms. This side-by-side association seems to be confined to a small subset of genes. Nonetheless, a minor component of the disrupted gene expression programme was more significantly altered in R6/1 “good” performers, probably indicating a homeostatic program in response to the mHtt insult that is more effectively activated in “good” mutant mice than in “poor” mutant mice. Other authors have pinpointed that certain transcriptional alterations compensate for inadequate expression levels of proteins (reviewed in^15^), including the transcription factor NF-Y (^29^ and Supplementary Fig. S2). In any case, whether these genes represent a new avenue for novel therapeutic approaches by promoting/enhancing their activity deserves further investigation.

The cause of transcriptional variations in the R6/1 strain remains to be elucidated. Enrichment analysis of transcription factor binding sites identified specific putative regulators of DEGs between R6/1 “poor” and “good” mice (Supplementary Fig. S4). These regulators include the following: (i) NF-κB for downregulated genes in “poor” performers, which is in agreement with the presence of *Nfkb2* (which encodes the p52 subunit) in the list; (ii) p53, which targets *Cdkn1a* and *Ddit4*, for downregulated genes in “good” performers^43^; and (iii) the serum responsive factor SRF in the upregulated genes in “poor” performers, which is in agreement with the enrichment of axonogenesis-related genes in the list and the newly proposed role for this transcription factor in dendritic growth^44^. Considering that these mice are isogenic (although we cannot entirely exclude genetic drifts due to the propagation of sporadic mutations, strain contamination, etc., in our colony^45^, a plausible explanation for the differentially affectation of the activities of these transcription factors among R6/1 mice involves epigenetics. Epigenetic dysregulation in HD has been extensively documented in previous works^46,47^ and can occur in early stages of the disease^20^. We speculate that slight environmental differences before isolation in individual cages (*in utero* conditions, endured social hierarchies established in the infancy, maternal care, sporadic changes in the germline), together with the inheritance of parental epigenetic patterns, may produce a subtle but significant impact on the manifestation of the disease in adulthood that may explain the results of Supplementary Fig. S1.

In humans, the large interindividual variability has likely impeded the identification of appropriate biomarkers based on gene expression. In line with these observations, we demonstrated that, even animal models with a highly controlled genetic background and controlled environmental factors show certain phenotypical variabilities that are associated with transcriptional variations. Whether these associations can be determined using the transcriptional profiles of peripheral tissues/fluids is important for designing novel strategies for biomarker screening.

## Materials and Methods

### Animals and behavioural testing

The transgenic B6.Cg-Tg(HDexon1)61Gpb/J strain (known as R6/1)^48^ was maintained on a pure C57BL/6 J background as previously reported^23^, under a 12-h light/dark cycle with food and water provided *ad libitum*. The transgene was transmitted through males. To reduce potential confounding factors from sex, only males were used in the study. Moreover, social hierarchy promotes interindividual differences^49,50^, therefore we isolated the subjects of study two weeks before behavioural testing assuming that the impact of social isolation affected uniformly across individuals and might be minimal in the short-term. For all behavioural tasks, transgenic mice and their wild-type littermates were analysed following the scheme shown in Fig. 1A starting at the age of 9 weeks during the light phase of the light/dark cycle: from 9:00 to 19:00 CET for Morris water maze, novel discrimination object and actimetry tasks, and from 9:00 to 13:00 CET for Rotarod and feet clasping measurements. Data were collected from three independent cohorts of males spanning a period of 10 months: 1^st^ cohort, n = 5 (wt) and 7 (R6/1), composed by 2 litters; 2^nd^ cohort, n = 10 (wt) and 10 (R6/1), composed by 4 litters; 3^rd^ cohort, n = 9 (wt) and 12 (R6/1), composed by 6 litters.

Spatial learning and memory were analysed by the Morris water maze, as previously described^51^ with minor modifications. The maze consisted of a round pool of water (0.95 m in diameter) divided into four virtual quadrants and surrounded by geometric cues on the walls. The escape platform was 2/3 cm under the water surface and was invisible to the mice. The water temperature was ~21 °C. During the acquisition phase, the animals performed 4 trials/day for 4 consecutive days, starting from each of the four quadrants in quasi random order. During the acquisition phase, the platform was located in quadrant 2. The time limit was 60 s/trial with a 10 min intertrial interval. If an animal did not reach the platform, it was manually placed on the platform for 10 s. The retention phase started the day after the completion of the acquisition phase and consisted of a single trial with no platform. A second retention phase was performed 72 hours after the end of the acquisition phase. The time required to locate the platform during the acquisition phase and the percentage of time spent in quadrant 2 during the retention phases were analysed using Smart software (Panlab, Spain).

Spontaneous locomotor activity was assessed by measuring the distance travelled by the mice in an opaque rectangular box (22 cm long × 44 cm width × 40 cm high) over 30 min. We also measured the % of time spent in the borders of the box (thigmotaxis). The novel object discrimination (NOD) task was carried out as previously described^52^ with minor modifications. On day 2, for habituation purposes, the animals were exposed to 2 objects (a yellow rhombohedron and a red cylinder) located in the centre of the box that were not used again during the test. The first sample trial was run on day 3. Each mouse was placed in the centre of the box, which contained 3 copies of a novel object (blue cylinders) arranged in a triangle-shaped configuration. The animals were allowed to explore for 5 min. After a delay of 30 min, the second sample trial was performed with 4 novel objects (red cones) arranged in a quadratic-shaped spatial configuration. The animals explored this environment for 5 min and were allowed to rest for 30 min. The animals then underwent the test trial with 2 copies of the object from sample trial 2 (the recent objects) placed in the same position as in the earlier trial, and two copies of the object from sample trial 1 (the familiar objects), one of which was placed in the same position as in the earlier trial (the old non-displaced object) and the other of which was placed a new position (the familiar displaced object). Integrated episodic memory for “what”, “where” and “when” was analysed as previously described^52^: “What” was defined as the difference in time spent exploring the familiar and recent objects, “where” was defined as the difference in time spent exploring the displaced and non-displaced objects and “when” was defined as the difference in the time spent exploring the familiar non-displaced and recent non-displaced objects.

The rotarod test was performed with LE8200 equipment (Panlab, Harvard Apparatus). On the first day, the animals were minimally habituated until they were able to stay on the rod at a fixed rotational speed of 4 r.p.m for > 4 min. To minimize memory consolidation, the mice were trained on the acceleration protocol on the same day after a minimum of 1 hour of rest. The acceleration protocol involved an increase of 1 r.p.m. every 8.33 s starting at 4 r.p.m (day 1). The latencies to fall off the rod (s) were averaged over four trials. The procedure was repeated one day and two weeks later (day 2 and day 15, respectively). The learning score was calculated as the difference in the latencies between day 2 and day 1, whereas progression in motor impairment was determined by calculating the difference in latencies between day 15 and day 2. Following the rotarod test, feet clasping was measured as the time for which the animal clasped its hindpaws while it was suspended by its tail for 5 min. Progression rate was calculated as the difference between week 13 (day 15) and 11 (day 1).

The experimental protocols were approved by the Comité de Ética de Experimentación Animal - Órgano Habilitado de la Universidad de Cádiz and authorized by the Dirección General de la Producción Agrícola y Ganadera de la Junta de Andalucía according to European and regional regulations.

### RNA extraction and RT-qPCR

Five days after the final test, the animals were sacrificed by cervical dislocation, and different brain areas (following the sequence of the cerebellum, hippocampus, striatum and prefrontal cortex) were immediately dissected and submerged in RNAlater (ThermoFisher) until processing.

Total RNA was extracted using TRIzol (ThermoFisher) and reverse transcribed into cDNA using the RevertAid First-Strand cDNA Synthesis kit (ThermoFisher). qPCR was performed on the Qiagen Rotor-Gene Q Detection system using PyroTaq EvaGreen qPCR Mix Plus (Cmb-Bioline). For all kits, the manufacturers’ recommendations were followed. Each independent sample was normalized to the level of *Gapdh*, as this gene was not altered in R6/1 brains^20,23^. The following primer pair sequences were obtained from previous works: *Gapdh* (5′-CATGGACTGTGGTCATGAGCC-3′, 5′-CTTCACCACCATGGAGAAGGC-3′), *Penk* (5′-GGCGTGCACACTGGAATGT-3′, 5′-TCCCAGATTTTGAAAGAAGGCA-3′), *Plk5* (5′-CACCTGTGTTTGCCTTTCCC-3′, 5′-TTTTGAGGTAAAGGGACAAGGTG-3′), *Itpka* (5′-TACACTCGGCTTTCGCATTG-3′, 5′-CAAAGACACGGGTCACTTGCT-3′), *Rin1* (5′-GCGGCTGCCAGAAGCTAGT-3′, 5′-CCTGGAACATGAGCTCTGAGC-3′), *Gabrd* (5′-ATTGGAGGTGCTCCTGTGAAT-3′, 5′-CCATGTTTGCCTCTGAGATAT-3′), *Pde10a* (5′-TAACAATGCGAGTTGCTTCC-3′, 5′-CAGCCACTTTTCCACAGTCTC-3′), *Nfya* (5′-ACAAGGGACGGTCACTGTGAC-3′, 5′-TTTGGATAGCAGGCACAGAGC-3′), *Bcl2* (5′-CACACCTGGATCCAGGATAAC-3′, 5′-GAGAAATCAAACAGAGGTCGCA-3′)^20,23,24^, *Gfap* (5′-GGACAACTTTGCACAGGACCTC-3′, 5′-TCCAAATCCACACGAGCCA-3′), *H2Q7/8/9* (5′-AGGAGCAGAATTACACATGCCA-3′, 5′-CGCCATGTTGGAGAGAGTGTAT-3′)^53^, *Arg1* (5′-TGGCTTGCGAGACGTAGAC-3′, 5′-GCTCAGGTGAATCGGCCTTTT-3′)^54^, *Bax* (5′-TGAAGACAGGGGCCTTTTTG-3′, 5′-AATTCGCCGGAGACACTCG-3′)^55^, *Scn4b* (5′-CATGGTTTAGTCCTCTGGCTTGG-3′, 5′-CACGGCCCACCACTGTATTG-3′)^56^, *Tac1* (5′-ATGAAAATCCTCGTGGCCGT-3′, 5′-GTTCTGCATCGCGCTTCTTT-3′)^57^, and *Mbd2* (5′-CCTTAGCAGTTTTGACTTCAGG-3′, 5′-TGGCAATGTTGTGTTCAGGT-3′)^58^. Other sequences were: *Hsp90b1* (5′-GATGATCTCCCCCTCAATGTT-3′, 5′-ATATTCGTGCCGAACTCCTTC-3′), *Nfyb* (5′-GACAGCTACGTGGAGCCTCTG-3′, 5′-AGTCCATCTGTGGCGGAGAC-3′) and *Trpc4* (5′-TGAGAAGGAAGCCAGAAAGCTTCG-3′, 5′-CCTTAACATTCTCCTCCGTCAAGCC-3′).

### DNA extraction and CAG repeat analysis

After the aqueous and organic phases were separated during the TRIzol procedure, the latter phase was used to isolate gDNA according to the manufacturer’s instructions. To remove any potential guanidine isothiocyanate contamination, the DNA pellet was resuspended in water, treated with phenol-chloroform-isoamylic alcohol and precipitated with sodium acetate and ethanol. Conventional PCR was performed with 75 ng of gDNA using BioTaq DNA polymerase (Bioline) introducing some modifications from other works^48,59^: 0.8 µM primers, 1.5 mM MgCl_2_, 2.4 mM dNTP mix and 10% DMSO. The program (1 cycle of 94 °C for 5 min, 35 cycles of 94 °C for 30 s, 64 °C for 30 s and 72 °C for 2 min, and 1 cycle of 72 °C for 10 min) was run on a GeneAmp PCR System 2700. The primers were 5′-FAM-ATGAAGGCCTTCGAGTCCCTCAAGTCCTTC-3′ and 5′-GGCGGCTGAGGAAGCTGAGGA-3′. Two microliters of each reaction was mixed with 1 µl of formamide and 0.5 µl of a molecular weight standard (Orange DNA size standard, MCLAB) and run on an Abi Prism 3100 analyser using NimaPOP-7 polymer (Nimagen). The result of capillary electrophoresis was analysed with GeneMapper (Applied Biosystems, ThermoFisher).

### Statistical analysis

SPSS software (IBM Statistics), Excel (Microsoft) and R-based stats package were used for statistical analysis. Mann-Whitney U tests were performed to compare any phenotypical trait or differential gene expression between genotypes. To correlate any phenotypical output with gene expression variation we calculated both the Spearman’s rank-order and Pearson’s coefficients; as they produced similar results, only the Spearman’s correlation coefficient is depicted in Fig. 2. A linear regression t-test contrasted whether the correlation coefficient was significantly different from 0, corrected for multiple testing with the Benjamini-Hochberg method.

For transcriptomics studies, mice were selected according to the phenotype exhibited at the age of 11 and 13 weeks using Z-scores ((value − average)/standard deviation). Animals were classified as “good” and “poor” performers based on (i) the number of phenotypical traits with positive and negative scores, respectively (Fig. 4A left panel), and (ii) the average value of the score across all the behavioural parameters (Fig. 4A right panel).

### RNA-seq and bioinformatics

Striatal RNA from classified mice were individually processed using the clean-up protocol of the RNeasy Mini Kit (Qiagen), which also included on-column DNase I treatment. This purified RNA was analysed for RIN integrity and values > 8.30 were obtained. DNA libraries were produced for mRNA using the TruSeq Stranded mRNA kit (Illumina) and subsequently sequenced using a NextSeq apparatus (Illumina) at the facilities of the Unidad de Genómica (Cabimer, Seville, Spain) in a 75-bp paired-end configuration. The resulting reads (31.6 M/sample on average) were mapped onto the mouse genome GRCm38/mm10 using TopHAT Alignment (v 1.0.1) software. The normalization of read counts and differential expression analysis was conducted using the DEseq2 package from Bioconductor^60^. DEGs were filtered with an FDR threshold of 0.05 (adjusted *P*-value). The RNA-seq data can be downloaded from the Gene Expression Omnibus (GEO) database using the accession number GSE135057.

For meta-analysis purposes, we employed whole transcriptome RNA-seq data of HD KI mice with different numbers of CAG repeats (Q20, Q80, Q92, Q111, Q140, Q175) at different ages (2, 6, 10 months old), as reported in the original publication (Supplemental Table 1 from^21^). After dividing the transcriptomes into bins of equal number of genes, we counted the number of genes among the top 684 DEGs compared to the Q20 striatum from our resulting lists, namely “R6/1 poor”, “R6/1 good” (defined in Fig. 4F,G) and “rest” (overlapping genes in pair-wise comparisons between the R6/1 and wild-type striatum after excluding DEGs between R6/1 “poor” and “good” mice).

Other additional bioinformatic tools were: Venny (http://bioinfogp.cnb.csic.es/tools/venny/), for identification of overlapping genes between multiple lists of genes; DAVID 6.8 (https://david.ncifcrf.gov/), for overrepresentation analysis of Gene Ontology (GO) terms related to biological processes; and Pscan (http://159.149.160.88/pscan/), for overrepresentation analysis of transcription factor binding motifs of the TRANSFAC database.

### Immunodetection and ChIP analyses

Western blot and ChIP were performed on brain tissues as previously^20^. We used the following antibodies: NF-Y_A_ (sc-10779X, Santa Cruz Biotechnologies), NF-Y_B_ (Pab 001, GeneSpin), H3 (ab176842, Abcam), and HRP-conjugated secondary antibodies (Sigma-Aldrich Química S.A.). Western blot quantifications were performed using ImageJ software. DNA from pooled mice was immunoprecipitated and quantified by qPCR to determine the percentage of immunoprecipitated DNA compared to input DNA using the following primers: *Hsp90b1/Grp94* CCAATbox (5′-CCAATGAACGTAGGCGCATA-3′, 5′-TTCGCTAGAAGCCGGAAGTG-3′), *Hspa1a* CCAAT box (5′-CCATAGCAACAGTGTCACTAGTAGCA-3′, 5′-GGGTTCACTGGAGAGTACGGATT-3′), *Rab5b* CCAAT box (5′-TCACCAACTAGCACAAGTGAGAGACT-3′, 5′-GCTACTTATCCCGTCAATCTTCAAG-3′)^30^, and *Hbb-bh1* (5′-GCAAGGTCCAGGGTGAAGAAT-3′, 5′-GGTGTGAGGTATAGAAGCTTGGAGAT-3′)^20^.

## Supplementary information

Supplementary Material

Supplementary Table 1
