# Supplementary material for: Transcriptional correlates of the pathological phenotype in a Huntington’s disease mouse model
Source: Sci Rep. 2019 Dec 10;9:18696. doi: 10.1038/s41598-019-55177-9 (PMC6904489; doi:10.1038/s41598-019-55177-9)
Supplement: Supplementary file 1 — Supplementary Material [file 41598_2019_55177_MOESM1_ESM.pdf]

Supplementary material to "Transcriptional correlates of the pathological phenotype in a Huntington's disease mouse model"

Andrea Gallardo-Orihuela, Irati Hervás-Corpión, Carmen Hierro-Bujalance, Daniel Sanchez-Sotano, Gema Jiménez-Gómez, Francisco Mora-López, Antonio Campos-Caro, Monica Garcia-Alloza, Luis M. Valor

Supplementary Figure S1. Phenotypical and gene expression variations across mouse cohorts. Distribution of mouse litter assignation in the plots of Fig. 2B and C. Orange, cohort 1; pink, cohort 2; green, cohort 3.

Supplementary Figure S2. Analysis of the transcription factor NF-Y in the R6/1 strain. *A*, RT-qPCR assays of cortical and striatal samples of R6/1 mice and their wild-type littermates showed a progressive reduction in *Nfya* levels, whereas the levels of *Nfyb* were unaltered; n = 7 for wild-type and n = 5 for R6/1. *B*, In contrast, Western blot assays showed a specific increase in the protein levels of NF-Y_A (normalized to histone H3 levels); n = 6 for wild-type and n = 5 for R6/1. *C*, While *Nfya* was upregulated, the NF-Y target gene *Hsp90b1* (also known as *Grp94*) was downregulated; n = 24 for wild-type and n = 29 for R6/1. *D*, The binding of NF-Y to the CCAAT box of its target genes (e.g., *Hsp90b1*) was not altered in the brains of R6/1 mice compared to the brains of their wild-type littermates. The results from the cortex and striatum are pooled; n = 4 pools of 3-4 animals per genotype. The data are expressed as mean ± s.e.m. *, $P<0.05$; **, $P<0.005$; Mann Whitney U-test.

Supplementary Figure S3. Markers of worse HD phenotype do not necessarily correlate with specific HD phenotypical traits. Upper panel, the expression of phenotype-related genes, namely *Gabrd* (gamma-aminobutyric acid type A receptor delta subunit), *Scn4b* (sodium voltage-gated channel beta subunit 4), *Pde10a* (phosphodiesterase 10A), *Tac1* (tachykinin precursor 1), *Mbd2* (methyl-CpG binding domain protein 2), *Nfya* (nuclear transcription factor Y subunit alpha), and *Trpc4* (transient receptor potential cation channel subfamily C member 4), across the four groups of animals (n = 4 each group). §, $P<0.05$ between R6/1 “poor” and “good” animals; Mann Whitney U-test. The data are expressed as the mean ± s.d. Lower panel, summary of the Spearman coefficient values showing the correlation between

phenotypical traits and gene expression levels (n = 29). Significant correlations (unadjusted $P < 0.05$; linear regression t-test) between *Gabrd* and rotarod performance.

Supplementary Figure S4. Predictive analysis of transcription binding sites. Lists of DNA motifs ($P<0.05$, Pscan) that were specifically enriched in each subset of genes associated with the R6/1 phenotype.

Supplementary Figure S1. Phenotypical and gene expression variations across mouse cohorts

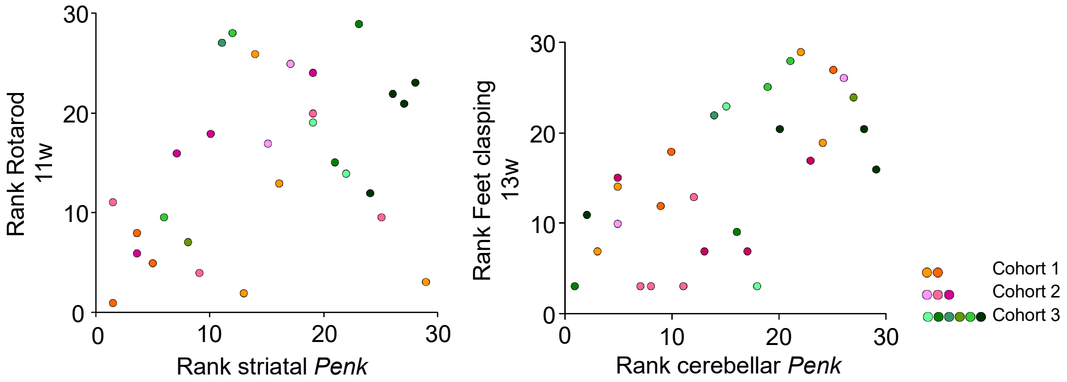

Supplementary Figure S2. Analysis of the transcription factor NF-Y in the R6/1 strain

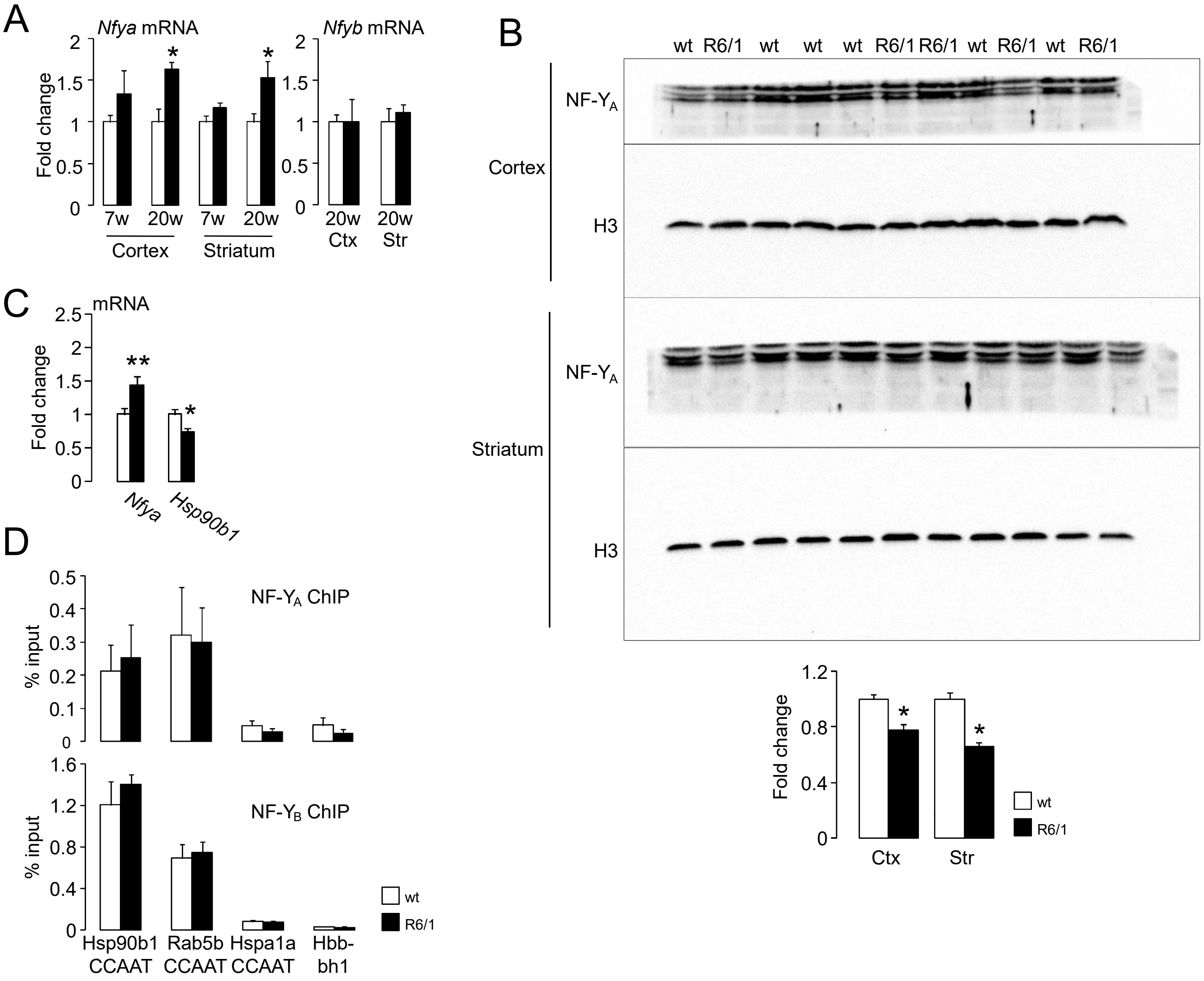

Supplementary Figure S3. Markers of worse HD phenotype do not necessarily correlate with specific HD phenotypical traits

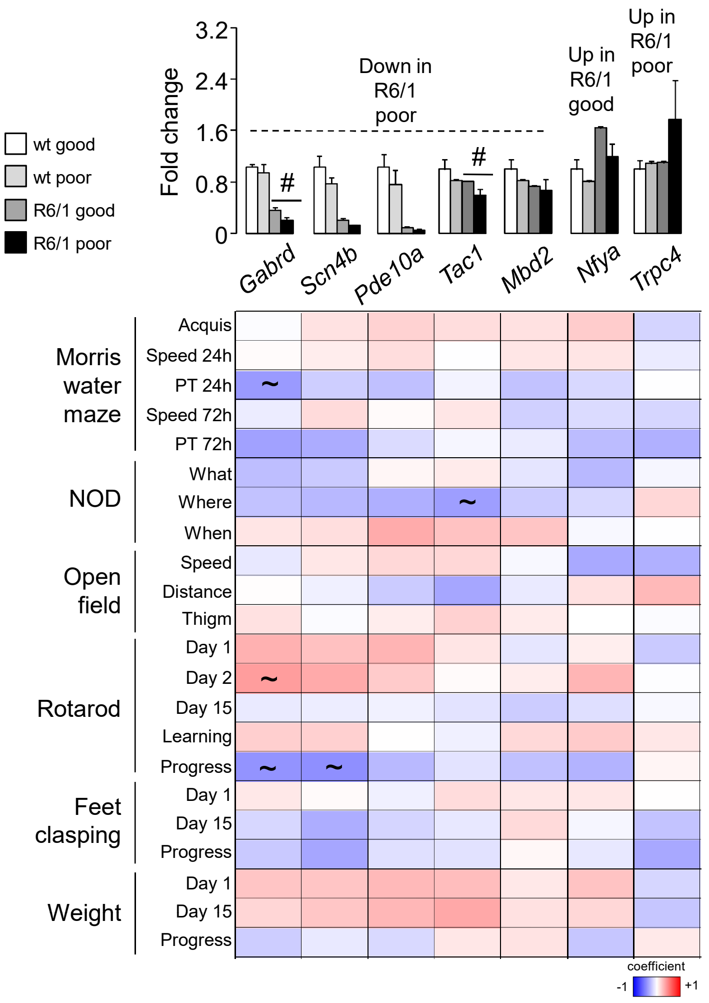

Supplementary Figure S4. Predictive analysis of transcription factor binding sites

TFBS in R6/1 poor DOWN	TFBS in R6/1 good DOWN	TFBS in R6/1 poor UP
V$LUN1_01	V$AP2REP_01	V$AHRARNT_02
V$MIF1_01	V$GATA1_01	V$ARNT_01
V$NFKAPPAB_01	V$HEN1_01	V$IK3_01
V$NFKB_C	V$MYOD_01	V$MYOD_Q6
V$NFKB_Q6	V$NRSF_01	V$P300_01
V$TAXCREB_01	V$P53_01	V$SRF_C
V$STAT3_02	V$STAT1_01	V$SRF_Q6
	V$ZIC1_01	
